# A clinico-pathological and molecular analysis reveals differences between solitary (early and late-onset) and synchronous rectal cancer

**DOI:** 10.1038/s41598-020-79118-z

**Published:** 2021-01-26

**Authors:** José Perea, Juan L. García, Luis Corchete, Sandra Tapial, Susana Olmedillas-López, Alfredo Vivas, Damián García-Olmo, Miguel Urioste, Ajay Goel, Rogelio González-Sarmiento

**Affiliations:** 1 Surgery Department, Fundación Jiménez Díaz University Hospital, 28040 Madrid, Spain; 2 Health Research Institute, Fundación Jiménez Díaz University Hospital, 28040 Madrid, Spain; 3 Molecular Medicine Unit, Department of Medicine, Biomedical Research Institute of Salamanca (IBSAL), Institute of Molecular and Cellular Biology of Cancer (IBMCC), University of Salamanca-SACYL-CSIC, 37007 Salamanca, Spain; 4 Digestive Cancer Research Group, 12 de Octubre Research Institute, 28041 Madrid, Spain; 5 Department of Surgery, 12 de Octubre University Hospital, 28041 Madrid, Spain; 6 Familial Cancer Clinical Unit, Human Cancer Genetics Program, Spanish National Cancer Research Centre (CNIO), 28029 Madrid, Spain; 7 Centro de Investigación Biomédica en Red de Enfermedades Raras (CIBERER), Instituto de Salud Carlos III, 28029 Madrid, Spain; 8 Department of Molecular Diagnostics and Experimental Therapeutics, Beckman Research Institute at City of Hope Comprehensive Cancer Center, Monrovia, CA 91016 USA

**Keywords:** Cancer, Genetics, Molecular biology

## Abstract

Rectal cancer (RC) appears to behave differently compared with colon cancer. We aimed to analyze existence of different subtypes of RC depending on distinct features (age of onset and the presence of synchronous primary malignant neoplasms). We compared the clinicopathological, familial and molecular features of three different populations diagnosed with RC (early-onset RC [EORC], late-onset RC, and synchronous RC [SRC]). Eighty-five RCs were identified and were evaluated according to their microsatellite instability, CpG Island Methylator Phenotype (CIMP) and chromosomal instability, as assessed by Next Generation Sequencing and microarray-based comparative genomic hybridization approaches. The results were subjected to cluster analysis. SRCs displayed the most specific characteristics including a trend for the development of multiple malignant neoplasms, a greater proportion of CIMP-High tumors (75%) and more frequent genomic alterations. These findings were confirmed by a clustering analysis that stratified RCs according to their genomic alterations. We also found that EORCs exhibited their own features including an important familial cancer component and a remarkable rate of mutations in *TP*53 (53%). Together, heterogeneity in RC characteristics by age of disease-onset and SRC warrants further study to optimize tailored prevention, detection and intervention strategies—particularly among young adults.

## Introduction

Colon cancer (CC) is the fourth most commonly diagnosed neoplasm worldwide, with cancers confined within the rectum (RC) being the eighth more frequent tumors. Collectively, colorectal cancer (CRC) is the third most common cancer worldwide, representing 11% of all cancers diagnosed^1,2^. The clinicopathological features of CRC can differ depending on the age of onset, the type of genomic alterations or the location of the tumor in the bowel, leading to different molecular subtypes which might benefit from a different clinical management^3–8^. Despite early-onset CRC (EOCRC) represents a rare entity, its incidence has steadily increased during the last couple of decades^9,10^. Likewise, data indicate a similar but a more alarming rise in the incidence of RC, which is projected to increase by as much as 270% by 2030^11^. At present, EOCRC represents 18% of all diagnosed RC^12^. Estimated numbers of new CRC cases by age in 2020 in the US show proportions of RC as follow: 36%, 35% and 24%, for individuals younger than 50 years of age, between 50 and 64, and older than 64 years, respectively^13^. No data have been published analyzing Synchronous CRCs.

Synchronous colorectal cancer (SCRC) represents a disease profile wherein more than one primary CRC are detected in a single patient at the time of diagnosis^14^. Several studies have demonstrated molecular concordance between tumors within the same individual although definitive results have not yet been achieved^15–19^. Indeed, our group recently published the possible clonal origin of a subset of SCRCs diagnosed in patients without hereditary forms of CRC^20^, and highlighting the significance of epigenomic patterns in the development of multiple primary neoplasms^21^. Because pathogenesis and clinical features largely depend on the location of the tumor in the colonic mucosa, a correlation between the hypothetical molecular basis of SCRC and the anatomical location in the colon has been proposed^22^.

Although some studies include RC within the left-sided tumors, we have recently confirmed the importance of defining three different locations for CRC (right side, left side and rectum) and the key-role of age at diagnosis^23^. Given the importance of anatomic location on CRC presentation as well as prognosis, together with distinct disease types, including early-onset rectal cancers (EORC) and synchronous tumors, we hypothesize that molecular and clinical patterns will vary among RC cases by age of disease-onset and synchronous malignant neoplasms. The purpose of our study was to define clinical, pathologic, demographic/familial and molecular patterns of RC by age of onset and among synchronous rectal tumors, and adding a clustering analysis as confirmation, in order to understand more deeply RC.

## Methods

### Families, samples and data collection

We defined three different subsets of RC at a single institution in Spain between January 2002 and December 2008 diagnosed: early-onset RC (EORC; individuals diagnosed with RC at an age < 50 years), late-onset RC (LORC [diagnosis at ≥ 69 years] and synchronous RC (SRC; 2 or more histologically distinct CRC identified in the same patient at the same time or in a period less than six months after the first diagnosis, with at least one of them located in the rectum). Cases with severely dysplastic tumors with ‘in-situ carcinoma’, hereditary polyposis and inflammatory bowel disease were excluded.

Demographic and clinicopathological information was obtained for each patient with EORC, LORC and SRC. Regarding SRC, tumor staging was defined by the tumor with the highest stage at diagnosis and tumor location was defined as previously reported^14^, and tumor relapse was defined either as regrowth at the anastomosis site (± 5 cm) or as the detection of new metastatic disease Family history of cancer (including at least three generations) and tumor tissue of paraffin-embedded samples were obtained from all patients. Regarding family history of cancer, all families were classified into four groups as we previously published^4^. All patients or a first-degree relative in the case of death of the index case provided written consent and the study was approved by the Ethics Committee of the “12 de Octubre” University Hospital. Disease-free survival (DFS) was defined as time from diagnosis to first event: disease recurrence or death from any cause. Recurrence risk, defined as the time from diagnosis to date of recurrence was also assessed. Recurrence diagnosis criteria included histological confirmation or radiologic evidence with subsequent clinical progression. Date of recurrence was defined as date of confirmatory imaging, or date of biopsy, as applicable. All cases had a complete colonoscopy at disease diagnosis, or if not possible (e.g. stenotic tumor), an intraoperative colonoscopic exploration. After primary surgical resection of the rectal tumor, patients underwent an endoscopic procedure within 9–12 months.

### Assesment of microsatellite instability (MSI) and CpG Island Methylator Phenotype (CIMP) status

Microscopic inspection of paraffin-embedded samples was performed by a pathologist. The acceptable proportion of tumor cells in the neoplastic material as well as the protocol for DNA isolation was previously reported^24^. We used the Bethesda panel to investigate the MSI status, considering MSI+ when 2 or more markers were altered. For the evaluation of CIMP, we studied the methylation status of the promoter regions of *CACNA1G*, *CDKN2A*, *CRABP1*, *IGF2*, *MLH1*, *NEUROG1*, *RUNX3* and *SOCS1* genes, and patients were categorized as CIMP-High, CIMP-Low or CIMP-0. The procedures for the evaluation and classification of MSI and CIMP statuses have been previously described^14^.

### Mutational status analysis by Next Generation Sequencing (NGS)

We used the Ion Torrent PGM platform with a commercial panel including 207 amplicons from 50 oncogenes and tumor suppressor genes (Supplementary Table S1). The protocols for the NGS library preparation, emulsion PCR, sequencing analysis, bioinformatics processing and data analysis were performed as previously reported^20^. For SRC cases, only rectal tumors were included for sequencing.

### Chromosomal instability

The analysis of copy number alterations (CNA) was carried out using two microarray-based comparative genomic hybridization (aCGH) platforms. Thus, from the total of 67 samples that could be satisfactorily processed, 17 were hybridized to the OncoScan Affymetrix array (SRC) and 50 (17 EORC, and 33 LORC) to the NimbleGen Human Whole Genome CGH array (Roche NimbleGen, Inc., Reykjavik, Iceland). Similar to NGS analysis, we excluded tumors not located in the rectum from the SRC group.

The protocol employed for the assessment of CNA for the NimbleGen platform has been previously described^24^. Data from these arrays were analyzed using the NimbleScan software (v.2.6) performing a spatial correction using the LOESS method, normalizing data values by qspline fit normalization and finally extracting the log2 ratio feature values. Both datasets have been included in the gene expression omnibus (GEO): for LORC (GSE108166) and EORC (GSE108220). The other 17 SRCs were analyzed using the OncoScan CNV FFPE Assay Kit according to the manufacturer’s instructions. Intensities from the OncoScan array were normalized and scaled using the ChAS console (v.4.0.0.385). Finally, weighted log2 ratio feature values were extracted for further analysis. The data have been recently described^20^ and have been also included in GEO database (GSE110026). In both platforms, log2 ratio data were segmented through the Piecewise Constant Fits segmentation method and then adjusted to reduce the outlier effect though Winsorization using the copy number package (v.1.22.0) in R (v.3.5.1). Segments from all the samples analyzed with both microarray platforms were combined using the intersect option of the bedtools toolset (v.2.17.0). The association between the three groups and the copy number status was assessed using the Fisher’s test in R through the function from the stats package (v.3.5.1). Multidimensional scaling was performed in the SIMFIT (v.7.5.1) statistical program using the Euclidean distance as the distance measure and the group average as the linkage method. Data coordinates retrieved from this plot were used to group samples through the MClust package (v. 5.4.5). The model with the best log-likelihood value was model VII (spherical with varying volume) with 5 components.

### Statistical analyses

Continuous variables were expressed as mean values plus/minus standard deviation (SD) and categorical variables were expressed as number of cases and their percentage. For the assessment of associations between age-of-onset and discrete variables either Pearson’s Chi Square (χ^2^) (parametric variables) or Fisher’s Exact Test (non-parametric variables) were used. Comparison of continuous variables was performed using Student’s t-test. Statistical analysis was performed using SPSS 17.0 (SPSS Inc., Chicago, IL, USA) and differences were considered statistically significant when *p* < 0.05. In order to identify potentially differentially altered regions for each population group, univariate analyses were carried out by performing an unconditional logistic regression for each candidate region. A false discovery rate (FDR) was calculated for each *p *value and regions with an FDR < 0.05 were considered statistically significant.

### Ethical approval

All procedures performed in studies involving human participants were in accordance with the ethical standards of the institutional and/or national research committee and with the 1964 Helsinki declaration and its later amendments or comparable ethical standards.

### Informed consent

Written informed consent was obtained from all individual participants included in the study or a first-degree relative in the case of death of the index case.

## Results

### Anatomoclinical and familial features

#### Global features

A total of 85 RCs was identified: 28 EORC, 37 LORC, and 15 SRC. Of these last 15 SRC patients, 5 had both tumors confined in the rectum (hence the total number of 85 tumors) and 10 had the RC paired with malignant neoplasms distributed in other colonic locations (3 in the right colon and 7 in the left colon (data not shown).

#### Comparative analysis between all the subset of rectal cancers

When we compared the three groups, SRCs exhibited the most different clinicopathological features. Overall, these patients were diagnosed at earlier tumor stages (75% in stage I; *p* < 0.001) and with a higher frequency of polyps (93%; *p* = 0.004), along with a higher mean of associated polyps (9.8; *p* = 0.004). Moreover, this subset of patients also possessed the highest rate of development of metachronous CRCs (MCRC) (33%; *p* < 0.001). As expected, the EORC group had the most frequent family history of cancer including Lynch-related and unrelated neoplasms with a concurrent lower frequency of polyps during follow-up (43%). Finally, the LORC group resembled EORC except for the higher sporadic component (92%; *p* < 0.001) and a higher association with the presence of polyps. Additional clinicopathological and familial characteristics of studied cases are shown in Table 1.

**Table 1:** Clinical, pathological and familial features of the patients included.

	EORC n (%)	LORC n (%)	SRC n (%)	*p* (χ^2^)
Patients	28	37	15	–
Mean age at onset ± SD (years)^a^	40.2 ± 4.9	78.5 ± 5.4	68.6 ± 10.6	–
**Gender**				
Male	18 (64)	30 (81)	11 (73)	NS
Female	10 (36)	7 (19)	4 (27)	
Mucin production^b^	5/21 (24)	4/34 (12)	3/9 (33)	NS
“Signet ring” cell histology^b^	2/21 (10)	2/34 (6)	1/9 (11)	NS
**Stage at diagnosis**^**b**^				< 0.001
I	8 (29)	2 (9)	15 (75)	
II	2 (7)	19 (16)	3 (15)	
III	8 (29)	6 (25)	2 (10)	
IV	10 (35)	10 (43)	0	
OS ± SD (months)^a^	44.6 ± 29.4	39.6 ± 22.5	57.5 ± 38.7	NS
DFS ± SD (months)^a^	36.7 ± 35.2	32.3 ± 34.5	60.5 ± 40.4	NS
Polyps development	12 (43%)	25 (67%)	14 (93%)	0.004
Average no. of polyps ± SD^a^	2.9 ± 9.5	2 ± 3.5	9.8 ± 9.9	0.004
**Type of polyps**				NS
Adenomatous	8 (66)	18 (72)	7 (50)	
Hyperplastic	2 (17)	3 (12)	0	
Serrated	0	0	0	
Mixed	2 (17)	4 (16)	7 (50)	
Metachronous CRC	0	1 (3)	5 (33)	< 0.001
**Family history of cancer**				
CRC	8 (29)	0	1 (7)	0.001
Aggregation for LR neoplasms	15 (54)	0	1 (7)	< 0.001
Aggregation for LUR neoplasm	9 (32)	3 (8)	1 (7)	0.02
Sporadic cases	11 (39)	34 (92)	13 (87)	< 0.001

^a^Statistical comparison was performed using Student’s t-test.

^b^Percentages shown are based on varying total numbers as some cases were excluded because only one biopsy was taken (stage D), or because tumors were severely dysplastic with “in situ” carcinoma and it was not possible to study any other characteristic.

DFS, disease-free survival; EORC, early-onset rectal Cancer; LR, lynch-related; LUR, lynch-unrelated; No, number; NS, not significant; LORC, late-onset rectal cancer; OS, overall survival; SD, standard deviation; SRC, Synchronous rectal cancer.

### Molecular analysis

#### Microsatellite instability (MSI) and CIMP

None of the studied patients presented with MSI. Regarding CIMP, adequate material for such analysis could only be obtained from 21 EORCs and 33 LORCs. Of these, 2 EORC (10%) and 12 LORC (36%) showed CIMP-High. Finally, 15 of the 20 SRC analyzed showed CIMP-High (75%).

#### Comparative analysis of mutational status

Sixty-five samples were successfully processed by NGS (33 LORC, 17 EORC and 15 SRC) to explore mutation patterns of RC across groups. Considering all tumors, the most frequent mutated genes were the *KRAS* (38%), *APC* (32%), and *TP53* (28%). Interestingly, the *KRAS* mutation rate was significantly higher in the SRC group (60%; *p* = 0.0048) whereas *TP53* showed the highest rate of mutation in EORC (53%; *p* = 0.012) across groups. It is also worth noting that *SMAD4*, which was differentially altered in the three populations, had a higher mutation frequency in SRC (20%; Table 2).

**Table 2:** Spectrum of cancer gene mutations among rectal cancer patients by age of disease-onset group and synchronous rectal cancer cases using next generation sequencing.

	Total		LORC (n = 33)		EORC (n = 17)		SRC (n = 15)		*p* (χ^2^)
	Cases	%	Cases	%	Cases	%	Cases	%	
KRAS	25	38.46	13	39.39	3	17.65	9	60	0.048
APC	21	32.31	11	33.33	4	23.53	6	40	NS
TP53	18	27.69	8	24.24	9	52.94	1	6.67	0.012
PIK3CA	6	9.23	2	6.06	2	11.76	2	13.33	NS
FBXW7	4	6.15	2	6.06	0	0	2	13.33	NS
NRAS	4	6.15	2	6.06	1	5.88	1	6.67	NS
SMAD4	4	6.15	1	3.03	0	0	3	20	0.036
SMARCB1	3	4.62	1	3.03	1	5.88	1	6.67	NS
BRAF	2	3.08	0	0	1	5.88	1	6.67	NS
CDKN2A	2	3.08	2	6.06	0	0	0	0	NS
GNAS	1	1.54	1	3.03	0	0	0	0	NS
ATM	1	1.54	0	0	0	0	1	6.67	NS

EORC, early-onset rectal cancer; LORC, late-onset rectal cancer; NS, not significant; SRC, synchronous rectal cancer.

#### Comparative analysis of chromosomal instability

A total of 67 samples were analyzed by aCGH. The most recurrently altered regions are shown in Supplementary Table S2. The group showing the highest number of genomic changes was SRC, where the most recurrent alterations were losses at 1q21 (65%) and 1p36 (59%), and gains at 8q24 and 20q13 (65%). The other two groups, on the contrary, seemed to be more heterogeneous; thus, losses at 9p13, 9q12 and 9q13 (all 41%) and gains at 19p13 and 19q11 (both 47%) were frequent in EORC, and gains at 9p13, 9q12, 19p12 and 19q11 (all 42%) were frequent in LORC. With respect to the altered regions occurring with statistically significantly different frequencies in the three groups, it is important to emphasize that most of them belonged to the SRC group, being gains at 8q24.3 and 20q13.12 (*p* = 0.001 and *p* = 0.004, respectively), and losses at 1q21.1 (*p* < 0.001), among others, those most notable (Tables 3 and 4).

**Table 3:** Gains occurring with statistically significantly different frequencies in the three groups.

Cytoband	LORC		EORC		SRC		*p* value	FDR
	Cases	%	Cases	%	Cases	%		
8q24.3	7	21.21	3	17.65	12	70.59	0.001	0.015
20q13.12	10	30.30	3	17.65	12	70.59	0.004	0.033
8q12	3	9.09	2	11.76	11	64.71	< 0.001	0.004
8q24	5	15.15	4	23.53	11	64.71	0.001	0.015
3q26	2	6.06	1	5.88	10	58.82	< 0.001	0.004
12p13.31	4	12.12	1	5.88	10	58.82	< 0.001	0.009
3q25.32	1	3.03	1	5.88	9	52.94	< 0.001	0.004
3q27.1	2	6.06	1	5.88	9	52.94	< 0.001	0.008
5p15.1	1	3.03	1	5.88	9	52.94	< 0.001	0.004
7q36.2	3	9.09	1	5.88	9	52.94	0.001	0.014
8q11.23	3	9.09	2	11.76	9	52.94	0.002	0.015
8q12.1	3	9.09	2	11.76	9	52.94	0.002	0.015
10q11.22	5	15.15	0	0.00	9	52.94	< 0.001	0.009
12p13.33	6	18.18	1	5.88	9	52.94	0.005	0.034
12p11.22	2	6.06	1	5.88	9	52.94	< 0.001	0.008
12p11.1	4	12.12	1	5.88	9	52.94	0.001	0.015
12q23.3	2	6.06	2	11.76	9	52.94	< 0.001	0.009

EORC, early-onset rectal cancer; FDR, false discovery rate; LORC, late-onset rectal cancer; SRC, synchronous rectal cancer.

**Table 4:** Losses occurring with statistically significantly different frequencies in the three groups.

Cytoband	LORC		EORC		SRC		*p* value	FDR
	Cases	%	Cases	%	Cases	%		
1q21.1	5	15.15	2	11.76	11	64.71	5.39E−04	0.040
1p36.23	5	15.15	0	0.00	9	52.94	3.76E−04	0.040
11q13.4	0	0.00	0	0.00	8	47.06	7.45E−06	0.005
15q25.2	0	0.00	1	5.88	7	41.18	1.09E−04	0.038
15q25.3	0	0.00	2	11.76	7	41.18	1.82E−04	0.040
15q26.1	0	0.00	3	17.65	7	41.18	2.47E−04	0.040
6p21.2	0	0.00	1	5.88	6	35.29	5.29E−04	0.040
11p15.4	0	0.00	1	5.88	6	35.29	5.29E−04	0.040
11p13	0	0.00	1	5.88	6	35.29	5.29E−04	0.040
11p12	0	0.00	1	5.88	6	35.29	5.29E−04	0.040
15q15.1	0	0.00	1	5.88	6	35.29	5.29E−04	0.040
15q21.1	0	0.00	2	11.76	6	35.29	8.22E−04	0.049
15q22.1	0	0.00	2	11.76	6	35.29	8.22E−04	0.049
15q25.2–q26	0	0.00	1	5.88	6	35.29	5.29E−04	0.040

EORC, early-onset rectal cancer; FDR, false discovery rate; LORC, late-onset rectal cancer; SRC, synchronous rectal cancer.

### Clustering analysis using MCLUST

**Figure 1:**
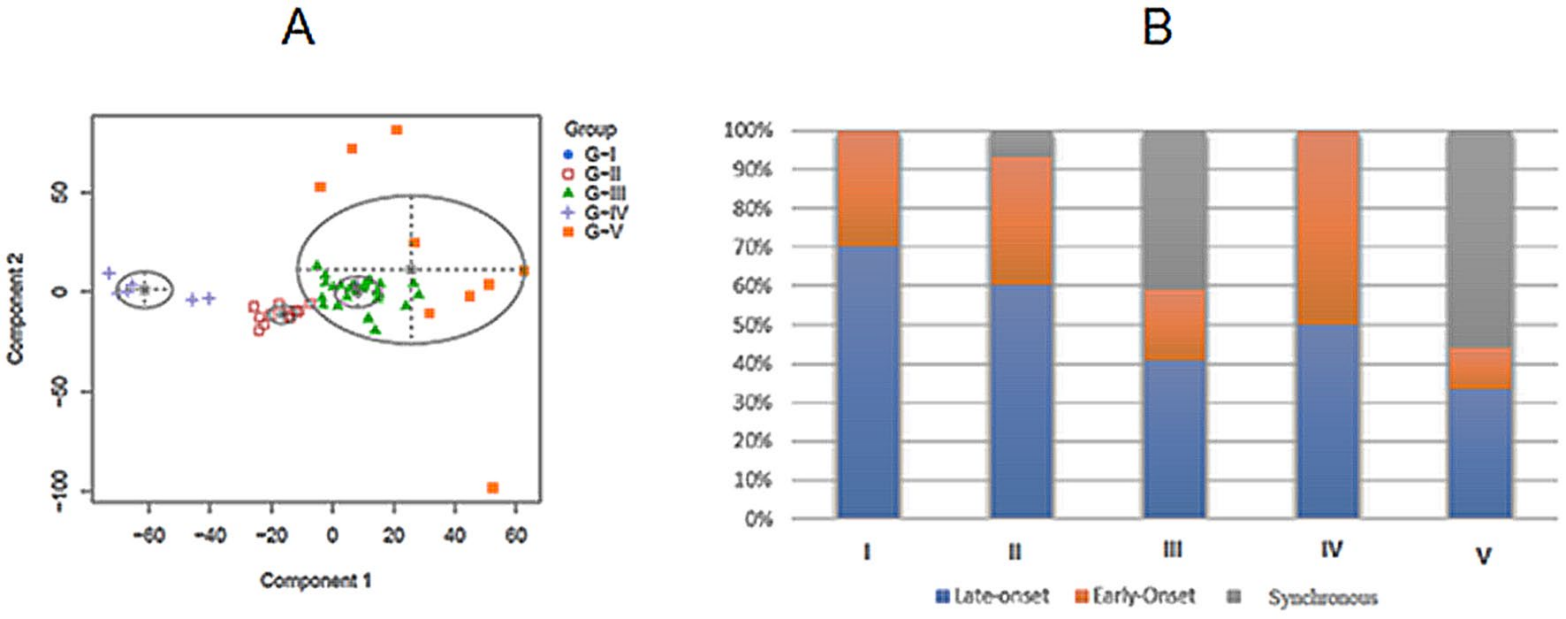
Genomic alteration clustering for rectal cancer cases. (**A**) Grouping of rectal cancers with the MClust method (https://mclust-org.github.io/mclust/). (**B**) Distribution of rectal cancers within the five groups from the MClust analysis.

RCs were clustered into five main categories according to their genomic alterations (Fig. 1). The first group (Group-I [G-I]) was composed of 10 cases (15% of all RCs) 70% of which corresponded to the LORC population. The second group (Group-II [G-II]) contained 15 cases (22% of all RCs) and was enriched in LORC (60%), even though it also included EORC cases (33%) and 1 patient with SRC (7%). The third group (Group-III [G-III]) was the largest one, which included 27 cases (40% of all RCs). In this group tumors were confined from the three studied populations: 40% of LORC, 40% of SRC and 20% of EORC. The fourth group (Group-IV [G-IV]) harbored 6 cases (9% of all RCs), with a similar representation of LORC and EORC. Finally, the fifth group (Group-V [G-V]) included 9 cases (13% of all RC) with most of them belonged to the SRC (56%) and only 33% and 11% having LORC and EORC, respectively.

There were no clinical differences between groups except for the distribution of SRC. Thus, all cases with both tumors confined in the rectum were categorized within G-III. Considering EORC and LORC collectively, 2 of the 5 RC patients that had synchronous high-degree dysplasia in other colonic locations were also included within G-III. Interestingly, from the 7 RCs showing synchronous cancers in organs other than the colon, the only 2 associated with digestive tract malignancies (gastric cancers) and were categorized within G-III. All tumors stratified within G-IV showed aneuploidy (defined as alteration-gain or loss-of whole chromosomes: at least a 50% of any arm of each chromosome) while this feature was present in 87%, 78%, 78% and 20% of G-II, G-V, G-III and G-I cases, respectively (Supplementary Table S3). With regards to genomic alterations, we observed a progressive increase as we moved from G-I to G-V (Supplementary Table S4). Accordingly, G-I exhibited the lowest percentage of genomic alterations (only 15% of the cases) as well as the lowest mutation rate obtained by NGS (Supplementary Table S5), whereas G-V was the group harboring the highest frequency of recurrent chromosomal alterations with gains at 3q26–q29 being the most prevalent. On the contrary, G-II and G-IV were very homogeneous groups in terms of number of recurrent alterations, prevailing gains at 19p and 19q in G-II, and gains at 7q, 16p, 19p, 19q and 20q in G-IV. Regarding G-III, it was a group with some specific alterations, although with low frequencies, which may be indicating its larger sporadic component. Recurrent chromosomal abnormalities with different percentage of affectation in each group are detailed in Table 5.

**Table 5:** Chromosomal segments frequently and differentially altered between the five groups from the MClust analysis.

	G-I	%	G-II	%	G-III	%	G-IV	%	G-V	%
Gains	18p11.32–p11.21	10	19p12	100	20q11.22–q13.33	44	7q11.21–11.23	100	3q27.1	89
	19p13	10	19q11–q13.11	100	9p13.1–9q12	41	7q22.1	100	3q26.1	78
	20q11–q13	10	19p13	93	20q11	41	16p13.12–p11.1	100	3q29	78
					20q13	41	19p13.3–q12	100	8q12.1	78
							19q13.12–q13.43	100	8q21.3	78
							20q11.21–q11.23	100	8q24.21	78
									8q24.22	78
									13q14.2	78
									13q22.1	78
Losses	19p13.11	10	3p12–p11	33	1q21	43	1p31.1	67	6q26–6q27	67
			1p32.3–p31.1	27	18p11.32	39			1p35–p34	56
			1p22.1–p21.1	27	18q11	39			3p21	56
					5q13	36			5q35	56
					8p23				8p12–p11	56
					18q12				10p15	56
					18q21–q23				11q12	56
									12q24	56
									17p13	56
									18q21–q23	56
									19p13	56
									19p12	56
									22q11–q13	56

Percents indicate the percentage of tumors with the chromosomal segment alteration within each group.

## Discussion

In contrast with the progressive decrease in diagnoses in older populations, the incidence of CRC in patients aged < 50 years has increased during the last two decades. In the context of EOCRC, the proportion of patients having cancer within the rectum seems to be experiencing an even more remarkable increase since RC represents up to 18% of all CRC diagnosed in young patients^12^. Several studies have highlighted the importance of the anatomical location in the pathogenesis and therapeutic responses suggesting that colorectal tumors should be evaluated as separate entities depending on the location of the lesion^25,26^. Moreover, some studies have focused specifically on RC subtypes and suggested that EORC may differ from LORC on a biological basis and in response to multimodality therapy^27^. In view of this premise and considering the high prevalence of RC in young adults, comparative analyses of rectal tumors are required as a suitable approach to go forward in the individualized management of RC.

From the three subsets of RCs studied in our series (EORC, LORC and SRC), SRCs presented with the most dissimilar characteristics—both from a clinical and molecular viewpoint. In comparison with the other groups, SRCs were characterized by an earlier age of diagnosis possibly because of the greater number of symptoms in these patients. In contrast, EORC was the group with the greatest delay in the diagnosis probably due to the low suspicion rate derived from the infrequency of CRC in young adults^28^. Another interesting point regarding the SRC group was its higher association with the development of polyps during the follow-up as well as with the development of MCRCs, what would be supporting the hypothesis that a field effect is operative in SCRC^29,30^. In this sense, also the large rate of CIMP-High tumors within this population (75%) might confirm the contribution of epigenetic alterations to field effects previously proposed^29,31^. These findings are also in accordance with our previous studies in which we concluded that CIMP+ tumors were more frequent in patients diagnosed with SCRC than in patients with an isolated CRC or MCRC^20,21^. Interestingly, we observed some molecular alterations that appeared to be linked with the SRC subset. Losses in 1q21 have been previously described in relation with EOCRC and gains in 8q24, a locus associated with CRC susceptibility polymorphisms which harbors *MYC*, an important proto-oncogene over-expressed in numerous tumors and related with the presence of synchronous adenomas elsewhere in the colon^24,32–34^. Finally, it is worth mentioning the locus 20q13 for being related with worse prognoses in RC^35^, and the locus 3q whose gains have been correlated with some types of neoplasia including anal cancers when there is association with human papillomavirus infection^36,37^.

In our series, the subset of RC diagnosed at an early age also showed some specific features such as a remarkable absence of MSI. In accordance with this, the EORC group revealed an absence of Lynch syndrome cases despite the familial cancer component underlying the group confirms this risk factor for the development of CRC even without a known hereditary component^38^. This group also displayed the higher rate of *P53* mutations and the lower rate of *KRAS* mutations. We have recently published the relationship between EOCRC and *P53* mutations in patients without associated polyps^39^ proposing that *P53* mutations may be an indicator of worse prognosis, in line with what has been said by other authors specifically for RC^40^. Regarding *KRAS* mutations, our findings point out that young population might need different therapeutic approaches, and corroborate the variability in the mutation rate of this gene as other studies according to the age and tumor location has also seen^41^.

Apart from the already known reality that RC may be understood as a different entity from CC, our findings suggest that there might be even different types of RC when criteria such as the age of diagnosis or the presence of multiple synchronous CRCs are considered. In this sense, in our series SRC appears to be different from other RCs. From a clinical point of view, SRC showed a higher tendency to develop malignant neoplasms in other colonic and extracolonic locations. Molecularly, SRC showed different molecular features which included a larger proportion of CIMP-High tumors as well as a greater number of genomic alterations, some of which seemed to be specific for this type of RC. Although EORCs also demonstrated their own features, they did in a less pronounced way, being the most striking findings their greater family component and the high rate of mutations in *P53*.

Despite the sample size of this study is limited, our findings may serve as a starting point for larger studies. Two points should be underlined regarding our findings: the persistent results that position SRC as a separate group of RC since these tumors were mainly confined within two of the five groups obtained by clustering analysis; and the higher possibility of developing future malignancies (including not only CRC but also other digestive tumors) in those patients with multiple primary neoplasia may serve as another good approach to this type of cases. Further studies with larger sample size would be recommended to confirm and substantiate our findings, especially regarding Synchronous rectal tumors.

## Supplementary information

Supplementary Information 1.
