# Supplementary material for: A clinico-pathological and molecular analysis reveals differences between solitary (early and late-onset) and synchronous rectal cancer
Source: Sci Rep. 2021 Jan 26;11:2202. doi: 10.1038/s41598-020-79118-z (PMC7838158; doi:10.1038/s41598-020-79118-z)
Supplement: Supplementary file 1 — Supplementary Information 1. [file 41598_2020_79118_MOESM1_ESM.docx]

a clinico-pathological and molecular analysis reveals differences between SOLITARY (early and late-onset) and synchronous rectal cancer

**Short title:** MOLECULAR PROFILES IN RECTAL CANCERS

Authors: José Perea, MD, PhD^1,2^; Juan L. García, PhD^3^; Luis Corchete PhD^3^; Sandra Tapial^4^; Susana Olmedillas-López PhD^2^; Alfredo Vivas, MD^5^; Damián García-Olmo, MD, PhD^1,2^; Miguel Urioste, MD, PhD^6,7^; Ajay Goel PhD, AGAF^8^; Rogelio González-Sarmiento, MD, PhD^3^.

**Affiliations:**

1) Surgery Department, Fundación Jiménez Díaz University Hospital, 28040 Madrid, Spain. damian.garcia@uam.es (D.G-O.).

2) Health Research Institute-Fundación Jiménez Díaz University Hospital, 28040 Madrid, Spain. susana.olmedillas@fjd.es (S.O-L.).

3) Molecular Medicine Unit-Department of Medicine, Biomedical Research Institute of Salamanca (IBSAL), and Institute of Molecular and Cellular Biology of Cancer (IBMCC), University of Salamanca-SACYL-CSIC, 37007 Salamanca, Spain. jlgarcia@usal.es (J.L.G.); [lacorsan@hotmail.com](mailto:lacorsan@hotmail.com) (L.C); gonzalez@usal.es (R.G.-S.).

4) Digestive Cancer Research Group. 12 de Octubre Research Institute, 28041 Madrid, Spain. stapialsantos@gmail.com (S.T.).

5) Department of Surgery. 12 de Octubre University Hospital. 28041 Madrid, Spain. Alfredovivas7@gmail.com (A.V.).

6) Human Genetics Group, Human Cancer Genetics Program, Spanish National Cancer Centre (CNIO), 28029 Madrid, Spain. murioste@cnio.es (M.U.).

7) Centro de Investigación Biomédica en Red de Enfermedades Raras (CIBERER). Instituto de Salud Carlos III, 28029 Madrid, Spain.

8) Department of Molecular Diagnostics and Experimental Therapeutics, Beckman Research Institute at City of Hope Comprehensive Cancer Center, Duarte, CA 91016, USA. ajgoel@coh.org (A.G.).

**Table S1. List of genes included in the commercial panel employed for the NGS analysis.**

| *ALB1* | *JAK2* |
| --- | --- |
| *AKT1* | *JAK3* |
| *ALK* | *IDH2* |
| *APC* | *KDR* |
| *ATM* | *KIT* |
| *BRAF* | *KRAS* |
| *CDH1* | *MET* |
| *CDKN2A* | *MLH1* |
| *CSF1R* | *MPL* |
| *CTNNB1* | *NOTCH1* |
| *EGFR* | *NPM1* |
| *ERBB2* | *NRAS* |
| *ERBB4* | *PDGFRA* |
| *EZH2* | *PIK3CA* |
| *FBXW7* | *PTEN* |
| *FGFR1* | *PTPN11* |
| *FGFR2* | *RB1* |
| *FGFR3* | *RET* |
| *FLT3* | *SMAD4* |
| *GNA11* | *SMARCB1* |
| *GNAS* | *SMO* |
| *GNAQ* | *SRC* |
| *HNF1A* | *STK11* |
| *HRAS* | *TP53* |
| *IDH1* | *VHL* |

**Table S2. Most recurrently altered regions for the three studied groups.**

|  | Gains | | | Losses | | |
| --- | --- | --- | --- | --- | --- | --- |
|  | **Region** | **cases** | **%** | **Region** | **cases** | **%** |
| LORC (n=33) | 9p13.1-p11 | 14 | 42 | 3p12 | 8 | 24 |
|  | 9q12-q13 | 14 | 42 | 3q11 | 8 | 24 |
|  | 19p12 | 14 | 42 | 5q13.2 | 8 | 24 |
|  | 19q11.q13.11 | 14 | 42 | 8p23.3 | 8 | 24 |
|  | 7q11.21-q11.23 | 13 | 39 | 18q22.3 | 8 | 24 |
|  | 20q11.1-q11.23 | 13 | 39 | 18q23 | 8 | 24 |
|  | 19p13.3-p13.11 | 12 | 36 |  |  |  |
|  | 1q21.1 | 11 | 33 |  |  |  |
|  | 13q34 | 10 | 30 |  |  |  |
|  | 20q12 | 10 | 30 |  |  |  |
|  | 20q13.11-q13.33 | 10 | 30 |  |  |  |
|  |  |  |  |  |  |  |
| EORC (n=17) | 19p13.3-p12 | 8 | 47 | 9p13-p11 | 7 | 41 |
|  | 19q11-q13.43 | 8 | 47 | 9q12 | 7 | 41 |
|  | 7q21.3-q22.1 | 6 | 35 | 9q13 | 7 | 41 |
|  | 7p22 | 5 | 29 | 9q21.11 | 6 | 35 |
|  | 7q11.23 | 5 | 29 | 10q11.21-q22 | 5 | 29 |
|  |  |  |  | 18q11.2-18q12.3 | 5 | 29 |
|  |  |  |  | 18q21.33-18q22.3 | 5 | 29 |
|  |  |  |  |  |  |  |
| SRC (n=17) | 8q24.13-q24.3 | 11 | 65 | 1q21 | 11 | 65 |
|  | 20q13 | 11 | 65 | 1p36 | 10 | 59 |
|  | 3q25-q27 | 10 | 59 | 1p35 | 9 | 53 |
|  | 8q12 | 10 | 59 | 18p11 | 9 | 53 |
|  | 12p13-p11 | 10 | 59 | 1p22 | 8 | 47 |
|  | 20q11-q13 | 10 | 59 | 1p13 | 8 | 47 |
|  | 5p15 | 9 | 53 | 4q21-q22 | 8 | 47 |
|  | 7q36 | 9 | 53 | 5q12.3 | 8 | 47 |
|  | 10q11 | 9 | 53 | 11q13 | 8 | 47 |
|  | 13q22-q34 | 9 | 53 | 18q11 | 8 | 47 |
|  | 17q25 | 9 | 53 | 189q21 | 8 | 47 |

EORC: Early-onset rectal cancer. LORC: Late-onset rectal cancer. SRC: Synchronous rectal cancer.

**Table S3. Categories from the MClust analysis.**

| RECTAL GROUP | CIMP | CATEGORY | ANEUPLOIDY | MUTATION | GAINS | LOSSES |
| --- | --- | --- | --- | --- | --- | --- |
| L | CIMP-High | I |  | *KRAS, PIK3CA, GNAS* | 18p11.32-p11.21 19p13 20q11-q13 (10%) | 19p13.11 (10%) |
| L | CIMP-Low/0 | I | +6, +13, +15 | *KRAS* |  |  |
| L | CIMP-Low/0 | I |  | *-* |  |  |
| L | CIMP-Low/0 | I |  | *-* |  |  |
| L | CIMP-Low/0 | I | -1, -2, -3, -4, -5, -9, -18 | *TP53* |  |  |
| L | CIMP-High | I |  | *-* |  |  |
| L | CIMP-Low/0 | I |  | *-* |  |  |
| E | CIMP-Low/0 | I |  | *-* |  |  |
| E | CIMP-Low/0 | I |  | *-* |  |  |
| E | CIMP-Low/0 | I |  | *-* |  |  |
|  |  |  |  |  |  |  |
| L | CIMP-Low/0 | II | +7, +19, +22 | *-* | 19p12,19q11-q13.11 (100%) | 3p12-p11 (33%) |
| L | CIMP-High | II | -1, -2, -3, -4, -5 | *-* |  |  |
| E | CIMP-Low/0 | II | -2, -3, -4, -5 , +7, +11, +12, +14, +15, +16, +19, +20, +22 | *TP53* |  |  |
| L | CIMP-High | II | 22 + | *NRAS* |  |  |
| L | CIMP-Low/0 | II | -1, -2, -3, -4, -5, -9 | *-* |  |  |
| L | CIMP-Low/0 | II | 19 + | *APC, KRAS, SMAD4, CDKN2A* |  |  |
| L | CIMP-Low/0 | II | +12, +19, | *-* |  |  |
| L | CIMP-Low/0 | II | 19 + | *-* |  |  |
| L | CIMP-High | II | -6, -18 | *APC, KRAS, TP53, FBXW7* |  |  |
| L | CIMP-High | II | 19+ | *APC, KRAS, CDKN2A* |  |  |
| E | CIMP-Low/0 | II |  | *APC, PIK3CA* |  |  |
| E | CIMP-Low/0 | II | 22 + | *PIK3CA, SMARCB1* |  |  |
| E | CIMP-High | II | +17, +19, +22 | *APC, KRAS* |  |  |
| E | CIMP-Low/0 | II | +17, +22 | *TP53* |  |  |
| S | CIMP LOW | II |  | *KRAS* |  |  |
|  |  |  |  |  |  |  |
| L | CIMP-Low/0 | III | -5, -6, -16, +20 | *TP53* | 20q11.22-q13.33 (44%) 9p13.1-9q12 (40%) | 1q21 (43%) |
| S | CIMP LOW | III | -4, +13 | *APC, KRAS, SMAD4* |  |  |
| S | CIMP LOW | III | +4, +7, +10, +13, +13, +16, -18, +20, +20 | *-* |  |  |
| S | CIMP HIGH | III |  | *APC, KRAS,* |  |  |
| S | CIMP HIGH | III |  | *TP53, ATM* |  |  |
| S | CIMP HIGH | III |  | *KRAS, PIK3CA* |  |  |
| S | CIMP HIGH | III | 13+ | *PIK3CA, NRAS, SMARCB1* |  |  |
| S | CIMP LOW | III | +8, +12, +13, +20 | *KRAS* |  |  |
| S | CIMP LOW | III |  | *-* |  |  |
| S | CIMP LOW | III |  | *APC, KRAS* |  |  |
| S | CIMP HIGH | III | -4 | *APC, KRAS, SMAD4* |  |  |
| S | CIMP HIGH | III | -4, -18 | *APC, KRAS, SMAD4* |  |  |
| L | CIMP-High | III | -18 | *APC, NRAS* |  |  |
| L | CIMP-Low/0 | III |  | *-* |  |  |
| L | CIMP-High | III | -5, -9, -12, +13, -15, -16, | *APC, KRAS, TP53* |  |  |
| L | CIMP-High | III | +19, +22 | *APC, KRAS, TP53* |  |  |
| L | CIMP-Low/0 | III | -1, -2, -4, -8, -11, +13, -14, -15, -18, +20, -21, -22 | *APC* |  |  |
| L | CIMP-High | III | -3, -4, -5, +6, +7, -9, +11, +13, +16, -18, +19, +20 | *APC, TP53* |  |  |
| L | CIMP-Low/0 | III | +2, +3, +4, +5 | *TP53* |  |  |
| L | CIMP-Low/0 | III | -16, -17, -19, -20, -22 | *-* |  |  |
| L | CIMP-Low/0 | III | +5, +7, -11, +12, +13, +14, -18, +21, +22 | *KRAS* |  |  |
| L | CIMP-Low/0 | III |  | *APC, KRAS* |  |  |
| E | CIMP-Low/0 | III | 13+ | *APC, TP53, NRAS* |  |  |
| E | CIMP-Low/0 | III | +3, -5, +6, +13,-14, -18 +20, +22 | *TP53* |  |  |
| E | CIMP-Low/0 | III | -5 +19 +22 | *KRAS, TP53* |  |  |
| E | CIMP-Low/0 | III | -11, -13, +16, +19 | *TP53* |  |  |
| E | CIMP-Low/0 | III | 13 | *-* |  |  |
|  |  |  |  |  |  |  |
| L | CIMP-Low/0 | IV | +19, +22 | *KRAS, FBXW7* | 7q11.21-11.23, 7q22.1, 16p13.12-p11.1, 19p13.3-q12, 19q13.12-q13.43, 20q11.21-q11.23 (100%) | 1p31.1 (67%) |
| L | CIMP-Low/0 | IV | -1, -2, -3, -4, -5, +7, -9, +13, -14, -18, +20, +21, +22 | *APC, KRAS* |  |  |
| L | CIMP-High | IV | -2, -4, -9, +19, +22 | *-* |  |  |
| E | CIMP-Low/0 | IV | -14, -15, -18, +19, -21 | *TP53* |  |  |
| E | CIMP-Low/0 | IV | -4, +17, -18, +20 | *APC, KRAS, TP53* |  |  |
| E | CIMP-Low/0 | IV | +17, +19, +22 |  |  |  |
|  |  |  |  |  |  |  |
| L | CIMP-High | V | -9, +13, -14, -17, +20, -21, -22 | *KRAS, TP53, PIK3CA, SMARCB1* | 3q27.1 (89%); 3q26.1, 3q29, 8q12.1, 8q21.3, 8q24.21, 13q (78%) | 6q26-6q27 (67%); 1p35-p34, 3p21, 5q35, 8p12-p11 (78%) |
| L | CIMP-Low/0 | V | +3, -19, -22 | *APC, KRAS* |  |  |
| L | CIMP-Low/0 | V | +7, +13, +14, +20 | *-* |  |  |
| E | CIMP-Low/0 | V | -11,+13, -14, -15 | *TP53, BRAF* |  |  |
| S | CIMP LOW | V | +6,-X | *BRAF* |  |  |
| S | CIMP0 | V | +12, -13, +14, +X | *FBXW7* |  |  |
| S | CIMP HIGH | V |  | *APC, KRAS, FBXW7* |  |  |
| S | CIMP HIGH | V |  | *PIK3CA, SMARCB1* |  |  |
| S | CIMP HIGH | V | +1, +2, +3, +5, +7, +8, +11, +12, +13, +20, +22, +X | *-* |  |  |

CIMP: CpG Island Methylator Phenotype. E: Early-onset rectal cancer. L: Late-onset rectal cancer. S: Synchronous rectal cancer.

**Table S4****. Genomic alterations in the five categories obtained from the MClust analysis.**

|  | **TOTAL CNAs** | **GAINS** | **LOSSES** |
| --- | --- | --- | --- |
| **Group I** | 1.5 (0-60) |  |  |
| **Group II** | 132 (29-2173) | 105 (0-1779) | 16 (0-478) |
| **Group III** | 314 (26-1983) | 166 (0-587) | 86 (0-1648) |
| **Group IV** | 1192 (680-1252) | 574 (376-709) | 455 (176-595) |
| **Group V** | 1168 (324-1687) | 746 (124-1100) | 335 (124-785) |

Numbers are expressed as median (minimum-maximum). CNAs: Copy number alterations.

**Table S5. Mutations found by NGS in the five categories obtained from the MClust analysis.**

|  | G-I |  | G-II |  | G-III |  | G-IV |  | G-V |  |
| --- | --- | --- | --- | --- | --- | --- | --- | --- | --- | --- |
|  | cases | % | cases | % | cases | % | cases | % | cases | % |
| KRAS | 2 | 20 | 5 | 33.33 | 12 | 46.15 | 3 | **50.00** | 3 | 37.5 |
| APC | 0 | 0 | 5 | 33.33 | 12 | **46.15** | 2 | 33.33 | 2 | 25 |
| TP53 | 1 | 10 | 3 | 20.00 | 10 | **38.46** | 2 | 33.33 | 2 | 25 |
| PIK3CA | 1 | 10 | 2 | 13.33 | 2 | 7.69 | 0 | 0.00 | 1 | 12.5 |
| FBXW7 | 0 | 0 | 1 | 6.67 | 0 | 0.00 | 1 | 16.67 | 2 | 25 |
| NRAS | 0 | 0 | 1 | 6.67 | 3 | 11.54 | 0 | 0.00 | 0 | 0 |
| SMAD4 | 0 | 0 | 1 | 6.67 | 3 | 11.54 | 0 | 0.00 | 0 | 0 |
| SMARCB1 | 0 | 0 | 1 | 6.67 | 1 | 3.85 | 0 | 0.00 | 1 | 12.5 |
| BRAF | 0 | 0 | 0 | 0.00 | 0 | 0.00 | 0 | 0.00 | 2 | 25 |
| CDKN2A | 0 | 0 | 2 | 13.33 | 0 | 0.00 | 0 | 0.00 | 0 | 0 |
| GNAS | 1 | 10 | 0 | 0.00 | 0 | 0.00 | 0 | 0.00 | 0 | 0 |
| ATM | 0 | 0 | 0 | 0.00 | 1 | 3.85 | 0 | 0.00 | 0 | 0 |

G: Group. NGS: Next generation sequencing.
